# The Study of Industrial Fatigue

**Published:** 1919-01-18

**Authors:** 


### THE STUDY OF INDUSTRIAL FATIGUE.

A strong Committee has been formed under the chairmanship of Professor Sherrington, F.R.S., for the study of this subject, and we would commend to it the inclusion in its labours of a study of industrial fatigue in nurses. In many branches of Labour, artizans have secured a working day of eight hours, with less on Saturday and none on Sunday—a working week of 54 hours, and in some trades agitations and even strikes are in progress for still shorter hours; but a nurse's working day is never less than ten hours, even when she has a colleague to relieve her of night duty. She may have one afternoon a week off duty, and an occasional Sunday, but in private nursing she is never secure of these, and in very many cases she is on duty both day and night, and gets her sleep when she can. She goes to bed at night, it is true, but in very many cases in private nursing she is liable to be called up, sometimes two or three times in the night, and for weeks together never has an uninterrupted night's sleep. At the end of some weeks of this, she goes back to the institution to which she belongs, and in times of epidemics may be sent off within an hour or two to another case.

This sweating of nurses is one of the matters that should engage the attention of the Royal College of Nursing. The College will be, if it is not already, a very powerful body in all that concerns nursing, and it might even now do worse than call the attention of Dr. Sherrington's Committee to industrial fatigue as it is manifested in overworked nurses.

